# Genome-wide association analysis identifies a susceptibility locus for sporadic vestibular schwannoma at 9p21

**DOI:** 10.1093/brain/awac478

**Published:** 2022-12-22

**Authors:** Katherine V Sadler, John Bowes, Charlie F Rowlands, Cristina Perez-Becerril, C Mwee van der Meer, Andrew T King, Scott A Rutherford, Omar N Pathmanaban, Charlotte Hammerbeck-Ward, Simon K W Lloyd, Simon R Freeman, Ricky Williams, Cathal John Hannan, Daniel Lewis, Steve Eyre, D Gareth Evans, Miriam J Smith

**Affiliations:** Manchester Centre for Genomic Medicine, St Mary's Hospital, Manchester Academic Health Sciences Centre (MAHSC), Manchester M13 9WL, UK; Division of Evolution, Infection and Genomics, School of Biological Sciences, Faculty of Biology, Medicine and Health, University of Manchester, Manchester M13 9PL, UK; Centre for Genetics and Genomics Versus Arthritis, Centre for Musculoskeletal Research, Faculty of Biology, Medicine and Health, Manchester Academic Health Science Centre, The University of Manchester, Manchester M13 9PL, UK; Division of Genetics and Epidemiology, Institute of Cancer Research, Sutton, London SM2 5NG, UK; Manchester Centre for Genomic Medicine, St Mary's Hospital, Manchester Academic Health Sciences Centre (MAHSC), Manchester M13 9WL, UK; Division of Evolution, Infection and Genomics, School of Biological Sciences, Faculty of Biology, Medicine and Health, University of Manchester, Manchester M13 9PL, UK; Division of Evolution, Infection and Genomics, School of Biological Sciences, Faculty of Biology, Medicine and Health, University of Manchester, Manchester M13 9PL, UK; Department of Neurosurgery, and Neuroradiology Manchester Centre for Clinical Neurosciences, Salford Royal NHS Foundation Trust, Manchester Academic Health Sciences Centre (MAHSC), Manchester M6 8HD, UK; Department of Neurosurgery, and Neuroradiology Manchester Centre for Clinical Neurosciences, Salford Royal NHS Foundation Trust, Manchester Academic Health Sciences Centre (MAHSC), Manchester M6 8HD, UK; Department of Neurosurgery, and Neuroradiology Manchester Centre for Clinical Neurosciences, Salford Royal NHS Foundation Trust, Manchester Academic Health Sciences Centre (MAHSC), Manchester M6 8HD, UK; Department of Neurosurgery, and Neuroradiology Manchester Centre for Clinical Neurosciences, Salford Royal NHS Foundation Trust, Manchester Academic Health Sciences Centre (MAHSC), Manchester M6 8HD, UK; Department of Otolaryngology, Manchester Royal Infirmary, Manchester Academic Health Sciences Centre (MAHSC), University of Manchester, Manchester M13 9WL, UK; Division of Cancer Sciences, Faculty of Biology, Medicine and Health, University of Manchester, Manchester M13 9PL, UK; Department of Otolaryngology, Manchester Royal Infirmary, Manchester Academic Health Sciences Centre (MAHSC), University of Manchester, Manchester M13 9WL, UK; Brain Tumour Biobank, Salford Royal NHS Foundation Trust, Manchester Academic Health Science Centre (MAHSC), Manchester M6 8HD, UK; Department of Neurosurgery, and Neuroradiology Manchester Centre for Clinical Neurosciences, Salford Royal NHS Foundation Trust, Manchester Academic Health Sciences Centre (MAHSC), Manchester M6 8HD, UK; Division of Cardiovascular Sciences, School of Medical Sciences, Faculty of Biology Medicine and Health, University of Manchester, Manchester M13 9PL, UK; Department of Neurosurgery, and Neuroradiology Manchester Centre for Clinical Neurosciences, Salford Royal NHS Foundation Trust, Manchester Academic Health Sciences Centre (MAHSC), Manchester M6 8HD, UK; Division of Musculoskeletal and Dermatological Sciences, School of Biological Sciences, Faculty of Biology, Medicine and Health, University of Manchester, Manchester M13 9PL, UK; Manchester Centre for Genomic Medicine, St Mary's Hospital, Manchester Academic Health Sciences Centre (MAHSC), Manchester M13 9WL, UK; Division of Evolution, Infection and Genomics, School of Biological Sciences, Faculty of Biology, Medicine and Health, University of Manchester, Manchester M13 9PL, UK; Manchester Centre for Genomic Medicine, St Mary's Hospital, Manchester Academic Health Sciences Centre (MAHSC), Manchester M13 9WL, UK; Division of Evolution, Infection and Genomics, School of Biological Sciences, Faculty of Biology, Medicine and Health, University of Manchester, Manchester M13 9PL, UK

**Keywords:** vestibular schwannoma, VS, sporadic VS, NF2, GWAS

## Abstract

Vestibular schwannomas are benign nerve sheath tumours that arise on the vestibulocochlear nerves. Vestibular schwannomas are known to occur in the context of tumour predisposition syndromes *NF2*-related and *LZTR1*-related schwannomatosis. However, the majority of vestibular schwannomas present sporadically without identification of germline pathogenic variants.

To identify novel genetic associations with risk of vestibular schwannoma development, we conducted a genome-wide association study in a cohort of 911 sporadic vestibular schwannoma cases collated from the neurofibromatosis type 2 genetic testing service in the north-west of England, UK and 5500 control samples from the UK Biobank resource. One risk locus reached genome-wide significance in our association analysis (9p21.3, rs1556516, *P* = 1.47 × 10^−13^, odds ratio = 0.67, allele frequency = 0.52).

9p21.3 is a genome-wide association study association hotspot, and a number of genes are localized to this region, notably *CDKN2B-AS1* and *CDKN2A/B*, also referred to as the INK4 locus. Dysregulation of gene products within the INK4 locus have been associated with multiple pathologies and the genes in this region have been observed to directly impact the expression of one another. Recurrent associations of the INK4 locus with components of well-described oncogenic pathways provides compelling evidence that the 9p21.3 region is truly associated with risk of vestibular schwannoma tumorigenesis.

## Teaser

Sadler *et al*. report the results of a genome-wide association study investigating genetic contributors to the risk of sporadic vestibular schwannoma development, and reveal a highly significant risk locus (9p21.3). With 911 cases and 5500 controls, this is the first comprehensive GWAS investigating sporadic vestibular schwannoma.

## Introduction

Vestibular schwannomas (VS) are benign tumours that develop on the vestibular portion of the vestibulocochlear nerve. Arising from Schwann cells of the myelin sheath surrounding nerves in the internal auditory canal, VS growth frequently causes hearing loss, tinnitus and disequilibrium in affected individuals. Less-frequent symptoms include headaches, vertigo, visual disturbance and facial nerve weakness.^1^ Annual incidence of VS has been previously estimated to range from 1 in 64 000 to 1 in 90 000.^2–4^ A more recent estimate of sporadic VS incidence is 3.0–5.2 per 100 000 person-years, equating to a lifetime prevalence that exceeds 1 in 500 persons.^5^ VS account for approximately 9% of all non-malignant central nervous system tumours.^6^

Vestibular schwannomas are known to occur within the context of tumour predisposition syndromes, neurofibromatosis type 2 (*NF2*)-related and non-*NF2*-related schwannomatosis.^7–9^ However, the majority of VSs occur sporadically in otherwise healthy individuals.^2^ No environmental factors have been robustly linked to risk of VS development, except for exposure to cranial radiotherapy,^10^ which occurs extremely infrequently in the general population. Some incidences of solitary VS appear to cluster within families.^11,12^ Moreover, that phenotype and clinical outcome variability between, and within, *NF2*-related and non-*NF2*-related schwannomatosis-affected families suggests the existence of genetic variants that modify VS risk and clinical course.^13,14^ It is hypothesized that the missing heritability and variable presentation observed in VS cases can be explained, at least in part, by common low-penetrance, small-effect-size inherited genetic variants.

The aim of this study was to identify novel genetic variants in association with VS risk by conducting a genome-wide association study (GWAS) in sporadic VS (sVS) patients, negative for germline pathogenic *NF2* variants. It is intended that variants identified in association with VS presentation are utilized for clinical risk prediction. Stratifying individuals based on genetic risk of VS would enable more personalized care management and prognosis, identifying optimal treatment strategies for patients. Identification of new genetic associations may also identify novel therapeutic targets for the treatment of VS.

## Materials and methods

### Subjects

A total of 911 sVS case lymphocyte DNA samples and 5500 control lymphocyte DNA samples comprised the study cohort. All case samples were ascertained through the highly specialized NF2 genetic testing service in the north-west of England, UK. Samples were collated from both the Manchester Centre for Genomic Medicine and Salford Royal Foundation Trust. Cases had presentation of sVS without family history and were negative for identifiable germline pathogenic *NF2* variants. Patients with germline pathogenic *LZTR1* variants were also excluded; however, *LZTR1* screening was not conducted in all case samples.

All control samples were obtained from the UK Biobank (UKBB) project. The UK Biobank is a large-scale biomedical database containing genotype and extensive phenotype data on half a million UK-based individuals between the ages of 49 and 60 years.^15^ Control samples were filtered to include participants with self-declared ethnicity as ‘White’. Individuals were excluded for the following International Classification of Diseases (ICD) 10 descriptions: ‘Benign neoplasms of cranial nerves’ (D33.3) and ‘Neurofibromatosis’ (Q85.0). Approximately 50 000 of the earliest UKBB samples were genotyped on the UK BiLEVE array. While this platform has similar single nucleotide polymorphism (SNP) coverage to the UK Biobank Axiom® Array, these samples were also excluded to maximise shared SNP coverage between cases and controls. With no ICD code specifically defined for VS, no case samples were ascertained from UK Biobank.

### Ethics

Ethical approval of the use of anonymized samples from the Manchester Centre for Genomic Medicine archive and the collection of blood samples from patients with informed consent was obtained from the North West 7–Greater Manchester Central Research Ethics Committee, IRAS ID: 36817, Rec Ref: 10/H1008/74.

### Genotyping

DNA was extracted from blood samples following conventional methods and quantified by Nanodrop 8000 Spectrophotometer (Thermo Fisher). Genotyping was conducted by two service providers. The first 475 case samples were genotyped by Oxford Genomics Centre. A further 436 case samples were genotyped by Yourgene Health. Both service providers use the same Axiom 2.0 Assay manual workflow on UK Biobank Axiom™ 96-well arrays. The UK Biobank Axiom™ Array contains 820 967 SNP and indel markers.

Array intensity data CEL files were imported into Axiom Analysis Suite v4.0.3.3 with the Axiom_UKB_WCSG.r5 library. Axiom Best Practice Genotyping Analysis Workflow, which uses an adapted version of the BRLMM-P algorithm and incorporates the R package SNPolisher, was applied for genotype clustering and evaluation of clustering quality. Quality of genotyping was considered for each individual sample, in addition to plate batches. As recommended by Axiom, samples with a Dish quality control (DQC) metric < 0.82 and call rate < 97% were considered to have failed genotyping. Quality metrics were calculated for each locus, based on call frequency, Hardy–Weinberg equilibrium (HWE), cluster separation, width and positioning. Probes meeting the defined quality metrics were exported in PED format. Genotypes are referenced against genome build GRCh37/hg19.

Case and control genotypes were subject to further QC filters prior to analysis. We considered only autosomal SNPs and SNPs were removed according to the following parameters: call rate < 98%, minor allele frequency (MAF) < 0.01 and HWE-departure (*P* < 1 × 10^−04^). Samples were excluded for call rate < 98% and heterozygosity deviating ±3 SD from the mean.

### Statistical analysis

Case and control genotype data were merged after sample and SNP QC; statistical analysis of relatedness and ancestry was conducted on the merged data set. Statistical analyses were largely conducted using R (v3.4.2) and PLINK (v1.9). Identity-by-descent (IBD) analysis was performed using KING (v1.9) software, calculating relatedness between each pair of individuals to identify duplicate or closely related samples (defined here as second-degree kinship or closer). Samples with the lowest call rates were removed in these related pairs. In our cohort analysis, one duplicate case sample was identified, in addition to second-degree or closer relatives in both cases (five pairs) and controls (four pairs).

Flashpca_x84-64 (v2.0) was used to calculate eigenvectors and perform principal component analysis (PCA). Merging of case and control data with HapMap 3 data identified outliers with non-Western European (CEU) ancestry (Supplementary Fig. 1). Linkage disequilibrium (LD) metrics were based on HapMap 3 recombination rate data. SNPs were pruned from the data set using PLINK and regions of high LD excluded. PCA was used to identify population stratification within study participants and the first three principal components of PCA analysis were associated with case–control status.

The McCarthy Group Haplotype Reference Consortium (HRC) checking tool (v4.2) was used to identify ambiguous SNPs and forward-strand align genotypes to the HRC and 1000 Genomes reference.

### Imputation

Phasing and imputation of non-typed SNPs and haplotype phasing of genotype data were conducted through the Michigan Imputation Server (minimac4 v1.5.7), Eagle (v2.3) and HRC r.1.1 2016 (GRCh37/hg19). Imputed data were annotated with National Centre for Biotechnology Information (NCBI) data, and duplicate and low-confidence SNPs (*r*² < 0.5) were removed using bcftools and vcftools.

### Association analysis

Association between individual SNPs and risk of VS was assessed using Cochran–Armitage linear regression under a frequentist additive effect model in SNPTEST (v2.5.2). Odds ratios (OR) and 95% confidence intervals (CI) were determined using a logistic regression model, conditioned on the first three principal components. SNPTEST output was filtered to include variants with a MAF > 1%. Summary statistics were uploaded to Functional Mapping and Annotation (FUMA) GWAS for data visualization and exploration.

### Investigation of rs1556516 genotype effect on vestibular schwannoma presentation age in patients with sporadic vestibular schwannoma

Kaplan–Meier survival analysis was performed, followed by a log-rank test, to assess if risk allele G at SNP rs1556516 conferred an increased risk of earlier age at VS presentation within cases from our combined cohort. From the 776 cases that passed GWAS quality filters, age at VS presentation information was available for 668 individuals. Cases were grouped by genotype, CC, CG, GG. Analysis was performed using GraphPad Prism version 9.1.2 for Windows (GraphPad Software, San Diego, CA, USA).

### Investigation of rs1556516 genotype effect on vestibular schwannoma presentation age in NF2 patients

A separate cohort of 186 NF2 patients was genotyped for SNP rs1556516 to assess if the risk allele G conferred an increased risk of earlier age at VS presentation. Germline DNA samples from *NF2*-related schwannomatosis patients were collated from the Manchester Centre for Genomic Medicine DNA archive. Patients included in the analysis had a known age at VS presentation and possessed *NF2* pathogenic variants considered to be mild or moderate in genetic severity.^16^ Patients with severe mutations, such as frameshift and nonsense mutations, were not included in analysis as the large effect size expected to be conferred by severe variants would be expected to mask the smaller effect size of our identified risk locus genotype.

Due to repetitive sequences surrounding lead SNP, rs1556516, no appropriate primer designs were available for direct characterization of rs1556516 in the cohort of NF2 patients. Primers were instead designed for proxy SNP rs1537371 (*r*² = 1), primer sequences available in Supplementary Table 1. Sequencing was conducted using BigDye Terminator v3.1 Cycle Sequencing Kit (Applied Biosystems) on ABI 3730xl DNA Analyser semi-automated sequencers. A Kaplan–Meier survival analysis, followed by a log-rank test, was conducted to test for an association between genotype and age at VS presentation. NF2 patients were grouped by genotype at SNP rs1537371, AA, AC, CC, with C being the risk allele in complete linkage with risk allele G at SNP rs1556516. Analysis was performed using GraphPad Prism version 9.1.2 for Windows (GraphPad Software, San Diego, CA, USA).

### URLs

UKBB, http://www.ukbiobank.ac.uk/; PLINK v1.9, https://www.cog-genomics.org/plink2/; McCarthy Group Checking Tool HRC/1000G (v4.2), https://www.well.ox.ac.uk/∼wrayner/tools/; KING v1.9, https://people.virginia.edu/∼wc9c/KING/manual.html; Flashpca_x86-64 v2.0, https://github.com/gabraham/flashpca; HapMap3, https://www.sanger.ac.uk/resources/downloads/human/hapmap3.html; bcftools, http://samtools.github.io/bcftools/bcftools.html; NCBI, https://www.ncbi.nlm.nih.gov; vcftools, https://vcftools.github.io/index.html; Michigan Imputation Server, https://imputationserver.sph.umich.edu/index.html; SNPTEST v2.5.2, https://www.well.ox.ac.uk/∼gav/snptest/; FUMA GWAS, https://fuma.ctglab.nl/; GraphPad Prism (v9.1.2) www.graphpad.com; Ensembl Variant Effect Predictor (VEP) https://www.ensembl.org/info/docs/tools/vep/index.html; GTEx Portal https://gtexportal.org/home/; HaploReg (v4.1) https://pubs.broadinstitute.org/mammals/haploreg/haploreg.php; EpiMap Repository http://compbio.mit.edu/epimap/.

### Data availability

GWAS summary statistics from this study will be made available via the EBI GWAS Catalogue website (https://www.ebi.ac.uk/gwas, accessed 31 August 2022) on publication of the study.

## Results

### Cohort analysis

We performed a GWAS in a cohort of sVS patients. Quality filters, ancestral and PCA was conducted. PCA using HapMap3 data showed that cases and controls were largely well matched for genetic ancestry (Supplementary Fig. 1). Our cohort comprised 911 cases of sVS and 5500 UKBB control samples. Following sample and SNP quality filtering, 870 cases, 5273 controls and 492 266 cohort-intersecting SNPs remained. After QC exclusion for IBD and ancestral outliers, 776 case and 5221 control samples were included in the association analysis.

### Association analysis

**Figure 1:**
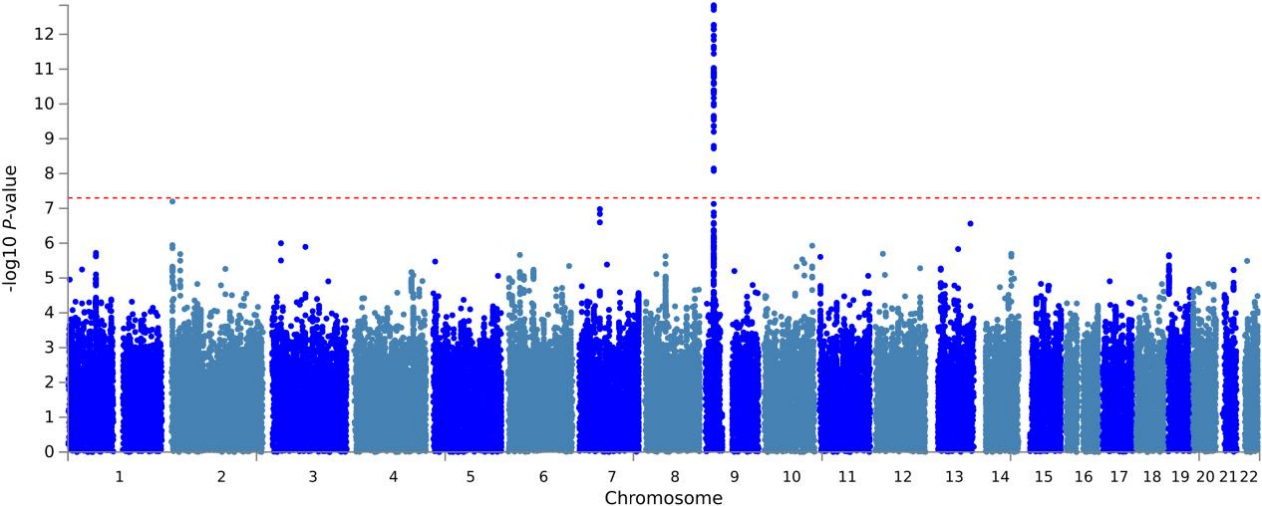
**Manhattan plot of MAF > 1% SNP *P*-values in the GWAS combined cohort across the autosomes**. The dashed red line represents *P*-value = 5 × 10^−8^.

Imputation of non-typed SNPs was conducted through the Michigan Imputation Server^17^ using the HRC panel. Association testing between each SNP and risk of VS was performed using a Cochran–Armitage linear regression test. Odds ratios and 95% CIs for SNPs with MAF > 1% were derived using a logistic regression model. Genome-wide, PCA-adjusted *P*-values for cohort association analysis are plotted in Fig. 1.

Our cohort analysis identified one genome-wide significant region (*P*-value < 5 × 10^−8^) in association with VS risk at 9p21.3, seen in Fig. 1. The lead SNP in this region of association was rs1556516 (*P* = 1.47 × 10^−13^, OR = 0.67, 95% CI: 0.60–0.75), mapping to intron 14 of *CDKN2B-AS1*. The OR values <1 at this locus suggest that the reference allele is the risk allele for the phenotype. Eleven SNPs are in linkage (*r*² = 1) with sentinel SNP rs1556516, and a further 40 are highly correlated (*r*² > 0.9), with a total of 54 SNPs possessed *P*-values < 5 × 10^−8^ within this region (Supplementary Table 2). Summary statistics of the lead SNP in the 9p21.3 locus can be seen in Table 1.

**Table 1:** Summary statistics of the genome-wide significant risk loci on chromosome 9p

SNP	Locus	Chromosome	Position	Other allele	Reference allele	MAF^a^	OR (95% CI)	β	SE	*P*-value	Annotated genes	Associated traits
rs1556516	9p21.3	9	22 100 176	C	G	0.477	0.672 (0.60–0.75)	−0.410	0.055	1.47 × 10^−13^	*CDKN2B-AS1*	Heart failure, parental longevity

MAF = minor allele frequency.

MAF is not risk allele frequency.

A number of genes are localized in the region of LD at the 9p21.3 risk locus surrounding rs1556516. In addition to *CDKN2B-AS1*, SNPs in high LD are also located in nearby tumour suppressor genes *CDKN2A* and *CDKN2B*, also referred to as the INK4 locus. A localized plot of SNP *P*-values at the 9p21.3 risk locus can be seen in Supplementary Fig. 2. *CDKN2B-AS1*, also referred to as *ANRIL*, is a long non-coding RNA (lncRNA) that downregulates *CDKN2B* expression when transcribed, through *cis*-acting heterochromatin formation.^18,19^*ANRIL* has been previously reported as a major GWAS hotspot,^20^ with multiple associations reported for cardiovascular phenotypes^21,22^ and neoplasms,^23^ including glioma.^24^*CDKN2A* encodes p16(INK4a) and p14(ARF); these gene products are translated from different reading frames of the *CDKN2A* gene and are therefore distinct proteins and not isoforms of one another. Protein p16(INK4a) acts as a negative regulator of cyclin-dependent kinases associated with cellular proliferation.^25^ Among other roles, p14(ARF) inactivates the protein MDM2, which is a negative regulator of tumour suppressor protein p53.^25^ Loss of p14(ARF) expression leads to an increase in MDM2-mediated degradation of p53, and germline mutations affecting p14(ARF) have been found to predispose carriers to multiple benign and malignant neoplasms.^26^ Interestingly, regulation of the p16(INK4a) locus has been associated with radiosensitivity in gliomas.^27^ It would be valuable to establish if this locus has a similar association in VS.

The second-strongest association was observed at 2p25.3 (rs73910511, *P* = 6.345 × 10^−08^, OR = 2.48), mapping to intron 2 of *EIPR1*. Three further SNPs are highly correlated with the sentinel SNP (*r*² < 0.8), rs116439544, rs7606684 and rs116430374, all positioned within intronic regions of *EIPR1*. *EIPR1* acts as a regulator of insulin secretion and distribution of mature dense-core vesicles.^28^ Dense-core vesicles are regulated secretory vesicles found in neurons and endocrine cells and are involved in the modulation of neurotransmission.^29^ Allele-specific methylation of *EIPR1* has been proposed as a mediator of psychiatric disorder susceptibility in phenotypically discordant monozygotic twins.^30^

The third-strongest evidence of association was identified at 7p11.2 (rs11238349, *P* = 1.05 × 10^−07^, OR = 1.33), positioned in intron 1 of *EGFR*. Two further SNPs are in high LD with lead SNP rs11238349 (*r*^2^ > 0.95), rs2302535 (*P* = 1.45 × 10^−07^, OR = 1.33) and rs12535578 (*P* = 2.52 × 10^−07^, OR = 1.32) both also positioned in intron 1 of *EGFR*. *EGFR* is an oncogenic transmembrane cell signalling protein involved in a range of cellular functions, including cell motility, differentiation, proliferation and survival.^31^ With described roles in lung cancer,^32^ breast cancer^33^ and glioma,^34^*EGFR* may also represent a VS predisposition gene.

A total of 37 genomic risk loci possessed *P*-values < 1 × 10^−8^, suggestive of association with the phenotype.^35,36^ A list of genomic risk loci with suggestive *P*-values is available in Supplementary Table 3.

### Investigation of rs1556516 genotype effect on vestibular schwannoma presentation age in patients with sporadic vestibular schwannoma

**Figure 2:**
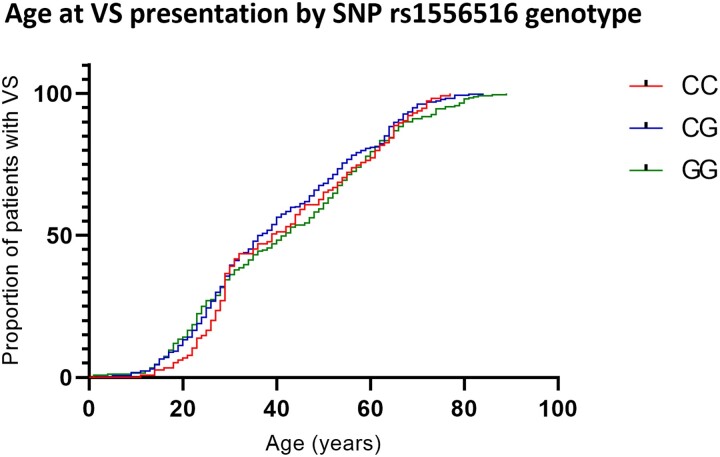
Kaplan–Meier survival curve of age at VS presentation in GWAS cohort of sporadic VS patients by genotype group at SNP rs1556516.

To assess if risk allele G at SNP rs1556516 conferred an increased risk of earlier age at VS presentation within cases we performed a Kaplan–Meier survival analysis, the results of which are shown in Fig. 2. The 668 cases with age at VS presentation data were grouped by genotype (CC = 115 cases, CG = 294 cases, GG = 259 cases). Age at VS presentation was plotted against the proportion of patients within the genotype group with VS presentation. A log-rank test found no significant difference between the survival curves (*P* = 0.1537). The mean (x̄) and interquartile range (IQR) of age at VS presentation in years were similar for each genotype group CC (x̄ = 42.3, IQR = 29.5), CG (x̄ = 40.2, IQR = 28), GG (x̄ = 42.4, IQR = 33.5).

### Investigation of rs1556516 genotype effect on vestibular schwannoma presentation age in *NF2*-related schwannomatosis patients

**Figure 3:**
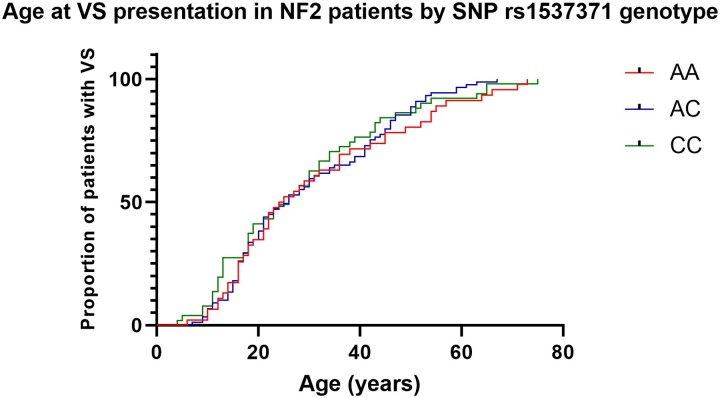
**Kaplan–Meier survival curve of age at VS presentation in *NF2*-related schwannomatosis patients by genotype group at SNP rs1537371**.

Due to repetitive sequences surrounding lead SNP, rs1556516, no appropriate primer designs were available for direct characterization of rs1556516 in a new cohort of *NF2*-related schwannomatosis patients. Primers were instead designed for proxy SNP rs1537371 (*r*² = 1). To assess if proxy risk allele C at SNP rs1537371 conferred an increased risk of earlier age at VS presentation in *NF2*-related schwannomatosis patients with low and mild severity *NF2* variants, we performed a Kaplan–Meier survival analysis and the results are shown in Fig. 3. One hundred and eighty-six cases of *NF2*-related schwannomatosis with age at VS presentation data were grouped by genotype (AA = 46 cases, AC = 89 cases, CC = 51 cases). Age at VS presentation was plotted against the proportion of patients within the genotype group with VS presentation. A log-rank test found no significant difference between the survival curves (*P* = 0.6564). The mean (x̄) and IQR of age at VS presentation in years were similar for each genotype group AA (x̄ = 31.0, IQR = 28), AC (x̄ = 29.7, IQR = 26), CC (x̄ = 28.5, IQR = 25.5).

## Discussion

We have conducted a primary GWAS identifying a susceptibility locus for sVS risk on chromosome 9p21.3 (*P*-value = 1.47 × 10^−13^) and have investigated this novel genomic risk locus in association with VS development.

*ANRIL* silencing has been associated with cell growth arrest and increased expression of the proteins encoded by *CDKN2A/B* within the INK4 locus.^37^ Deletion and deregulation of the INK4 region adjacent to *ANRIL* has been previously associated with multiple cancer types^38,39^ and somatic loss of heterozygosity (LOH) of the locus has been observed frequently in tumour samples, including neurofibromatosis type 1 (NF1) plexiform neurofibromas.^40–42^ Mouse models developed with knockouts of the different proteins encoded within the INK4 locus exhibit predisposition to spontaneous tumour development in comparison the their wild-type litter mates.^43^

*ANRIL* has also been implicated in the dysregulation of inflammatory genes, including *IL6* and *IL8.*^37^ Inflammatory stimuli, such as tumour necrosis factor-α (TNF-α) and interleukin-1β (IL-1β), activate transcription factor nuclear factor-κB (NF-κB), which has been demonstrated to interact directly with the promoter of *ANRIL*, inducing transcription.^37^ Pro-inflammatory cytokines, including IL-1β and IL-6, have been demonstrated to exhibit increased expression in human VS tissue compared to normal vestibular nerve samples.^44^ Moreover, TNF-α expression has been observed in, and associated with, the proliferation of Schwann cells, which may act in an autocrine model of cell signalling.^44^ It is also hypothesized that the expression of pro-inflammatory cytokines in the tumour microenvironment results in the recruitment of inflammatory cells, such as macrophages, causing increased VS tumour growth.^45,46^

NF-κB signalling has been observed to modulate drug response in lung cancers associated with activating *EGFR* mutations, in which increased NF-κB expression is associated with resistance to epidermal growth factor receptor (EGFR) tyrosine kinase inhibitor drugs.^47^ Constitutive signalling of EGFR is associated with activation of a number of oncogenic signalling cascades, including the PI3K/AKT/mTOR and RAS/RAF pathways.^48^ RAS has been found to act as a positive regulator of gene products in the INK4 locus, and homozygous deletion of the *CDKN2A* (INK4a/ARF) locus is a frequent finding in melanoma tumours, many of which harbour *NRAS* or *BRAF* mutations.^25^ The gene product of *NF2*, merlin, is a negative regulator of NF-κB activity^49^ and performs an inhibitory role in the PI3K/AKT/mTOR pathway,^50^ which can be modulated *via* RAS signalling.^51^ The recurrent observations of component dysregulation within these overlapping pathways has also been similarly described in glioblastoma,^52^ and provides compelling evidence that the 9p21.3 risk locus is truly associated with VS tumorigenesis.

To investigate the potential mechanism of action between variants in the associated 9p21.3 locus and risk of VS presentation, we employed a number of *in silico* resources to characterize further genomic features of the region. The 54 SNPs of genome-wide significance (*P* < 5 × 10^−8^) were input through the Ensembl VEP.^53^ Eight regulatory features overlapped the 54 variants, composing three enhancers and five promoter flanking regions. Additionally, four transcription factor binding motifs are found in proximity to SNPs rs10811656 and rs4977575.

HaploReg^54^ tool was used to explore annotations of SNPs linked with lead SNP rs1556516 in European (EUR) populations. Fifty SNPs were identified in high LD (*r*^2^ 0.8) with rs1556516, located in either intronic regions of *CDKN2B-AS1* or positioned downstream of the gene. Ten of the linked SNPs sit within known protein binding sites, and variants in 37 SNPs are predicted to alter regulatory motifs. Many of the linked SNPs have been previously reported in association with a number of disease phenotypes in the NHGRI-EBI GWAS Catalogue. In general, cardiovascular-related phenotypes such as aneurysms, artery calcification, myocardial infarction and strokes appear to be associated with the alternative allele genotype, while other phenotypes, such as asthma, endometriosis and haemorrhoidal disease are recorded in association with the reference allele genotype.

Utilizing the online GTEx portal,^55^ lead SNP rs1556516 was found to overlap a known splicing quantitative trait locus (sQTL) in pituitary tissue. Variant genotypes at the rs1556516 locus have been found to significantly affect the intron-excision ratio at this region (*P* = 0.0000017), with a reduced ratio in individuals homozygous for the risk allele G.

Use of the EpiMap repository^57^ suggests that *CDKN2B-AS1* is expressed in neural tissue, such as neurospheres and the brain. However, expression is not consistent across neural tissue types and it is unclear as to whether the available studied tissue types are relevant in the investigation of VS because Schwann cells have not been directly sampled, yet VS occur exclusively in Schwann cells.

The genomic annotations of promoters, enhancers, other regulatory features and previous GWAS associations within the 9p21.3 risk loci bolsters our hypothesis that non-coding variants within the region can result in mechanistic effects that impact clinical phenotypes, including risk of VS.

While there are currently no published GWAS investigating VS risk, a preprint article released in June 2021 by Shringarpure *et al.*^56^ outlines a large-scale GWAS investigating a number of self-reported rare disorders, including VS. In this study, 1216 cases with self-reported VS presentation and 168 029 controls were obtained from 23andMe, Inc. One genome-wide significant hit was identified in association with VS on chromosome 9p, lead SNP rs7341786 (*P* = 1.4 × 10^−15^, OR = 1.395) positioned in *CDKN2B-AS1*. This finding was validated in a UK Biobank case cohort selected using the phenotype ‘Benign neoplasm of cranial nerves’. The self-reported nature of cases in the discovery cohort and non-specific phenotyping in the validation set leaves this study vulnerable to non-specific associations. Moreover, known VS predisposing conditions, *NF2*-related schwannomatosis and *LZTR1*-related schwannomatosis, were not used as exclusionary criteria for cases. However, within our study SNP rs7341786 also reached genome-wide significance (*P* = 4.46 × 10^−11^, OR = 0.70). As we present our findings in respect to the reference sequence we report an OR < 1 as the reference allele G was found to confer risk of VS. As the preprint article has not been critically reviewed and published we cannot employ it for validation of our findings. However, replication of significant association at *CDKN2B-AS1* suggests the lead risk locus within our study is genuine.

Our investigation into the effect of lead associated SNP rs1556516 on VS presentation age in sporadic VS patients found no significant difference between genotype and age at VS presentation (Fig. 2). This suggests that the increased risk of VS conferred by SNP rs1556516 is not age-dependent and may be influenced by other variables such as genotypes at other loci or environmental stimuli. SNP rs1556516 may confer an increased risk to subtle dysregulation of a single component within the highly complex aforementioned oncogenic pathways. As multiple regulatory networks often exist within signalling pathways^50,51^ it is reasonable to assume that variants within them do not always result in linear outcome correlations. Moreover, it is possible that rs1556516 represents a proxy SNP for an uncharacterised risk variant in high LD with this marker.

Our investigation into the effect of proxy SNP rs1537371 on VS presentation age in *NF2*-related schwannomatosis patients found no significant difference between genotype and age at VS presentation (Fig. 3). Lack of an observable association may be due to the relatively small effect size the risk allele is expected to confer on VS risk, in comparison to *NF2* pathogenic variants of mild/moderate severity. Additionally, the cohort of 186 *NF2*-related schwannomatosis patients used in this age analysis is likely too small to detect the subtle effects of our identified risk loci. Sample collection for this analysis was constrained by the availability of genomic DNA samples and the number of patients with an identified *NF2* pathogenic variant defined as mild/moderate genetic severity. While no association between genotype and age at VS presentation in *NF2*-related schwannomatosis patients was detected, there does appear to be a slight enrichment in *NF2*-related schwannomatosis patients presenting with VS < 20 years in the homozygous CC risk allele group (Fig. 3); however, this increase is not significant.

While our cohort size (911 cases) may be considered a limitation of the study, power calculations suggest that 1000 cases would provide over 80% power to detect an effect with an odds ratio of 1.2, assuming an allele frequency of 20%. As risk allele G at lead SNP position rs1556516 has an allele frequency of approximately 50% and a predicted effect with an odds ratio of 0.67, detection of a significant genotype–phenotype association is possible in a cohort of this size. As sVS is a relatively rare disorder, sample availability is limited and therefore the prospect of conducting an independent validation association analysis, in the UK, is limited. Performance of replication GWAS in sVS requires collaboration with other specialized institutions internationally to form a validation cohort.

In conclusion, we have identified a susceptibility locus for sporadic VS at chromosome 9p21. Gene products within this region have demonstrated oncogenic roles, with pathway associations linked to VS tumorigenesis. Further investigation of the 9p21.3 locus in somatic VS samples may represent an interesting line of future work. LOH of the region encompassing *NF2* is a frequent observation in somatic VS samples but does not account for all cases.^58^ It is possible that LOH of the 9p21.3 locus may represent a mutational hit in VS tumours without observed *NF2* loss. While we did not detect an association between the 9p21.3 susceptibility locus genotype and VS age at presentation within this cohort, the region may act as a risk modifier in other patient groups. Future work to delineate the effect of this region on VS risk may be performed in *NF2*-related schwannomatosis patients, exploring a potential association between genotype at the 9p21.3 locus and VS presentation age.

## Supplementary Material

awac478_Supplementary_Data
