# Supplementary material for: Genome-wide association analysis identifies a susceptibility locus for sporadic vestibular schwannoma at 9p21
Source: Brain. 2022 Dec 22;146(7):2861–8. doi: 10.1093/brain/awac478 (PMC10317144; doi:10.1093/brain/awac478)
Supplement: awac478_Supplementary_Data [file awac478_supplementary_data.zip › brain-2022-01721-File006.pdf]

Supplementary material

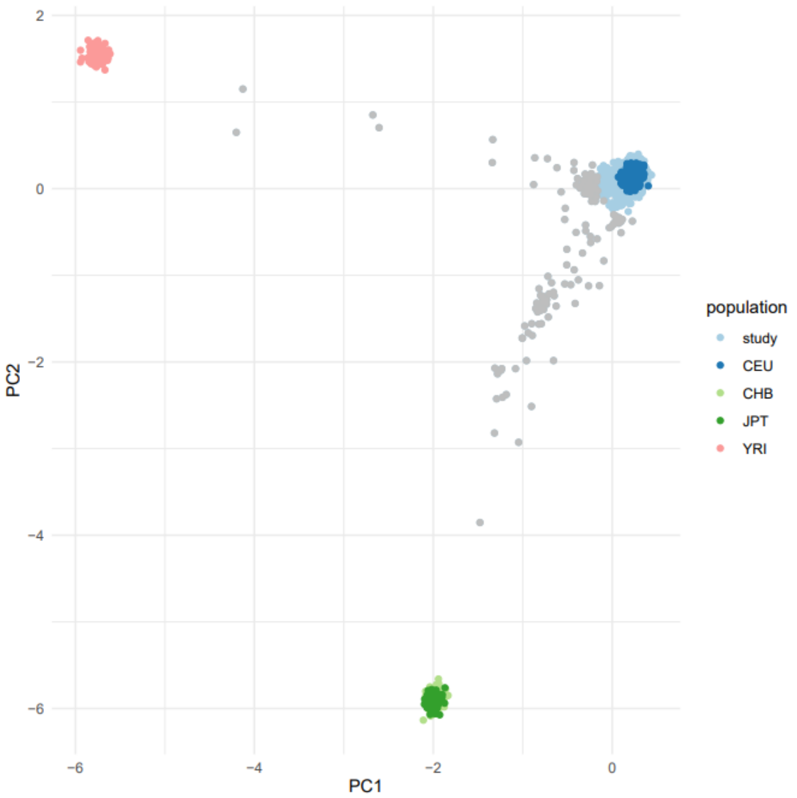

Supplementary figure 1. Combined cohort case population analysis using HapMap3 data. Grey and light blue markers indicate study samples in the combined analysis. Grey samples were deemed ancestral outliers and were excluded from downstream association analysis.

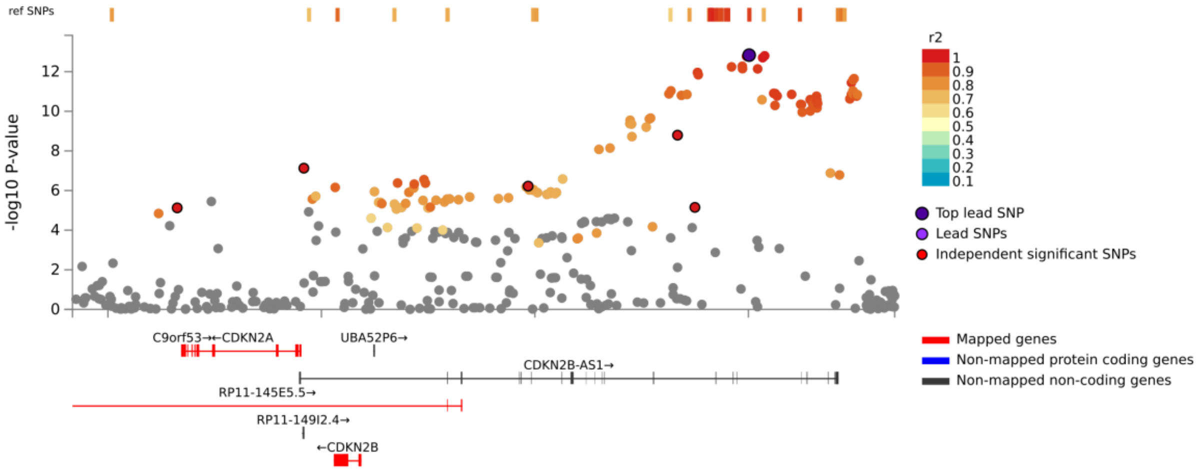

Supplementary figure 2. Localised plot of SNP *P*-values in the combined association analysis at the 9p21.3 risk locus.
