# Supplementary figures and images for: Genome-wide association analysis identifies a susceptibility locus for sporadic vestibular schwannoma at 9p21
Source: Brain. 2022 Dec 22;146(7):2861–8. doi: 10.1093/brain/awac478 (PMC10317144; doi:10.1093/brain/awac478)

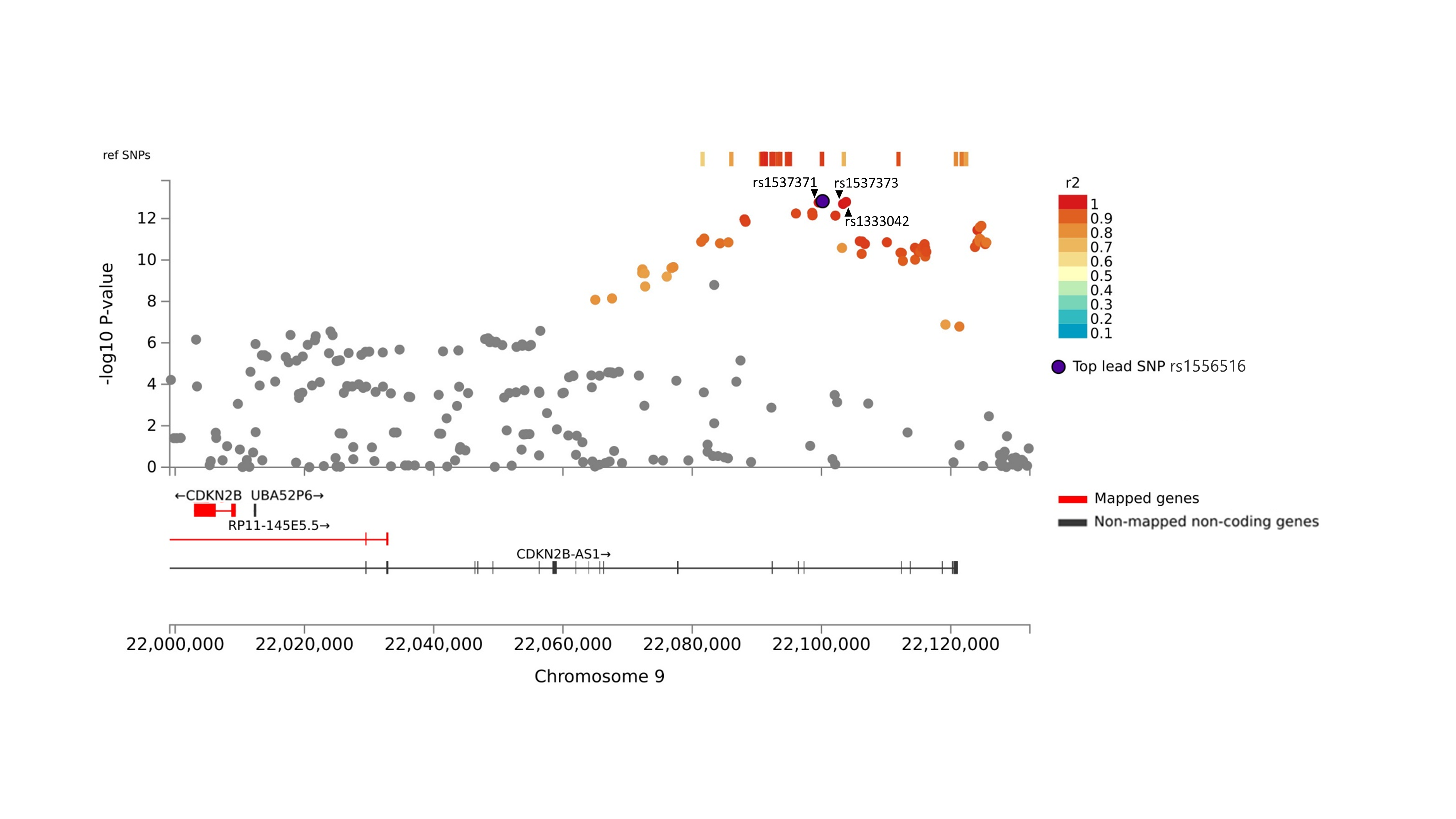

Supplement: awac478_Supplementary_Data [file awac478_supplementary_data.zip › brain-2022-01721-File007.tif]
